# Atrial overdrive pacing in drug-refractory postinfarction electrical storm case report

**DOI:** 10.1097/MD.0000000000046384

**Published:** 2026-05-12

**Authors:** Xin-Yuan Xu, Rui-Xia He, Hai-Jun Wang, Peng Liu, Guo-Li Ma, Li-Na Ji, Ba-Ya-Er Qi, Xiao-Ping Liu

**Affiliations:** a Department of Cardiology, Ordos Clinical College of Inner Mongolia Medical University, Ordos Central Hospital, Ordos, Inner Mongolia Autonomous Region, China.

**Keywords:** acute myocardial infarction, atrial overdrive pacing, cardiac resynchronization, electrical storm, ventricular arrhythmia

## Abstract

**Rationale:**

Drug-refractory electrical storm (ES) following acute myocardial infarction (AMI) constitutes a critical therapeutic challenge. Atrial overdrive pacing (AOP) provides physiological rhythm control by suppressing ventricular ectopy through synchronized atrioventricular activation, circumventing ventricular pacing-associated hemodynamic compromise.

**Patient concerns:**

A 62-year-old female with AMI developed recurrent polymorphic ventricular tachycardia/ventricular fibrillation refractory to antiarrhythmics (amiodarone/esmolol/lidocaine) and revascularization, fulfilling ES criteria.

**Diagnoses:**

The patient was diagnosed with acute anterior wall myocardial infarction, Killip class II–III acute left heart failure, secondary hepatic dysfunction, hypertension, type 2 diabetes mellitus and ES.

**Interventions:**

X-ray-guided temporary AOP (95 bpm) via subclavian access was implemented with concurrent β-blocker/sacubitril-valsartan optimization and electrolyte correction. Antiarrhythmics were discontinued post-AOP.

**Outcomes:**

Sustained arrhythmia suppression was achieved (0 recurrences/9 months), alongside improved left ventricular ejection fraction (40%→46%) and 70.7% N-terminal B-type natriuretic peptide precursor reduction (33,593→9839 pg/mL). Implantable cardioverter-defibrillator was declined without clinical sequelae.

**Lessons:**

AOP demonstrates dual therapeutic efficacy in refractory post-AMI ES: (1) physiological conduction restoration suppressing ectopic triggers and (2) avoidance of ventricular pacing-induced dyssynchrony. Fluoroscopic guidance ensures lead stability, positioning AOP as a hemodynamically favorable intervention in ES management.

## 1. Introduction

According to the 2017 AHA/ACC/HRS Guideline the electrical storm (ES) commonly defined as the occurrence of ventricular arrhythmias 3 or more times within 24 hour (separated by intervals of at least 5 minutes), each requiring intervention for termination.^[1]^ ES is a frequently encountered complication following acute myocardial infarction (AMI), is conventionally managed through revascularization strategies and antiarrhythmic pharmacotherapy. However, the management of recurrent ES refractory to both percutaneous coronary intervention and optimized medical regimens remains clinically challenging. In such scenarios, temporary atrial overdrive pacing (AOP) may demonstrate therapeutic potential. Given the limited literature on the application of this therapeutic approach for such disorders, we were motivated to document the case demonstrating its successful outcome.

## 2. Case presentation

A 62-year-old female with a 5-year history of suboptimally controlled hypertension and type 2 diabetes mellitus. No familial history of coronary heart disease and arrhythmia was reported. Presented with progressive exertional dyspnea (NYHA class II–III) and angina-equivalent chest pain over 6 months, acutely exacerbated 48 hours prior to admission. Initial vital signs demonstrated normothermia (36.0°C), sustained sinus tachycardia (130 bpm), stage II hypertension (139/100 mm Hg), and mild hypoxemia (SpO_2_ 93% on room air). Cardiopulmonary examination revealed jugular venous distension, bilateral basal crackles, and a pathological S3 gallop rhythm. No other significant comorbidities were documented.

**Figure 1.**
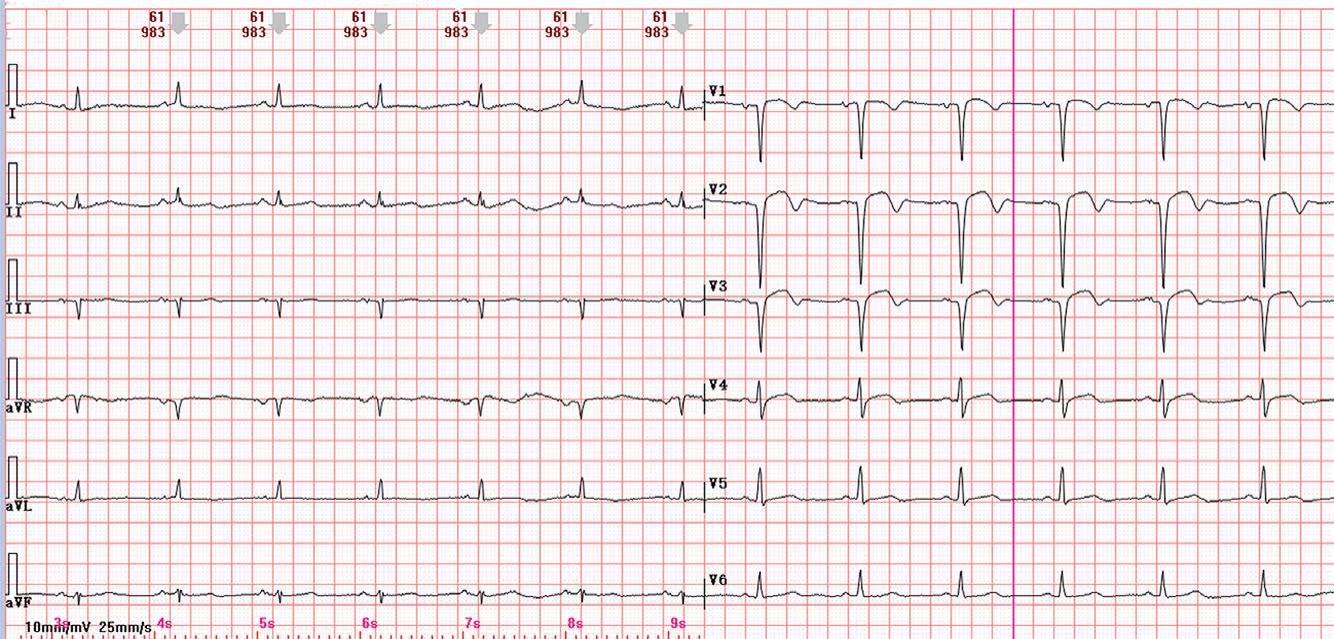
Twelve-lead ECG showing sinus rhythm, ST-segment elevation, abnormal Q wave, T-wave inversion in leads V1–V3, and low-voltage QRS complexes in limb leads. ECG = electrocardiogram.

The diagnostic evaluation, based on the patient’s 6-month history of exertional dyspnea and angina-equivalent symptoms, confirmed acute coronary syndrome complicated by acute decompensated heart failure and secondary hepatic dysfunction. Admission 12-lead electrocardiogram demonstrated sinus rhythm with anteroseptal ST-segment elevation (V1–V3), pathological Q waves, T-wave inversions, and low-voltage QRS complexes in limb leads (Fig. 1). Transthoracic echocardiography revealed severe anterior wall motion abnormalities (hypokinesia/akinesia), left ventricular dilatation (end-diastolic diameter 58 mm), and reduced systolic function (left ventricular ejection fraction 40%). Radiographic evaluation identified pulmonary interstitial edema on chest X-ray and bilateral pleural effusions via thoracic ultrasound. Laboratory analysis demonstrated marked elevation of cardiac biomarkers: high-sensitivity troponin T (hs-TnT) 1.34 µg/L (reference < 0.014 µg/L) and N-terminal B-type natriuretic peptide precursor (NT-proBNP) 33,593 pg/mL (reference < 125 pg/mL). Hepatic derangement was evidenced by alanine aminotransferase 1699 U/L (13–35) and aspartate aminotransferase 2349 U/L (7–40). Electrolyte profiling showed venous potassium 4.41 mmol/L (3.5–5.3) and magnesium 1.05 mmol/L (0.75–1.02), excluding significant ionic disturbances.

The definitive diagnoses included acute anterior wall myocardial infarction (MI),^[2]^ Killip class II–III acute left heart failure, secondary hepatic dysfunction, hypertension, and type 2 diabetes mellitus. Given the post-MI interval exceeding 48 hours with symptom resolution at admission, coupled with electrocardiographic evidence of established Q waves, deferred revascularization was planned. Pharmacological management commenced with dual antiplatelet loading (Aspirin 300 mg + Clopidogrel 300 mg), followed by maintenance therapy (Aspirin 100 mg daily + Clopidogrel 75 mg daily). Guideline-directed heart failure therapy included Sacubitril/Valsartan (50 mg twice daily) and cautious β-blocker initiation with Metoprolol Tartrate (6.25 mg twice daily), subsequently up-titrated to 12.5 mg twice daily on day 3 following hemodynamic tolerance confirmation. Adjunctive therapies encompassed hepatic protection (Silibinin Meglumine 140 mg 3 times daily and Polyenylphosphatidylcholine 456 mg 3 times daily), decongestive strategy (Torsemide 10 mg daily, Spironolactone 40 mg daily and Recombinant Human Brain Natriuretic Peptide, rhBNP 0.01 µg/kg/min continuous infusion), metabolic optimization (Dapagliflozin 10 mg daily), electrolyte management (Potassium Magnesium Aspartate 2 tablets 3 times daily), and Pitavastatin (2 mg daily) was introduced following hepatic function stabilization.

**Figure 2.**
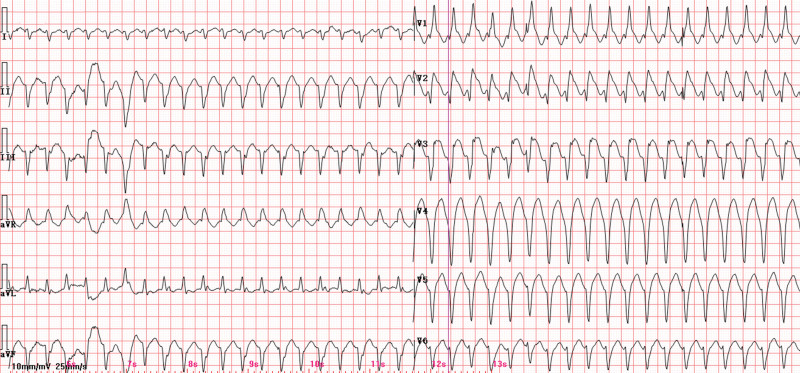
Ventricular tachycardia (VT).

**Figure 3.**
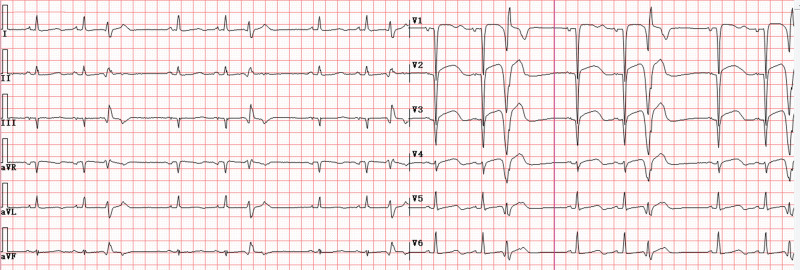
R-on-T premature ventricular contractions.

**Figure 4.**
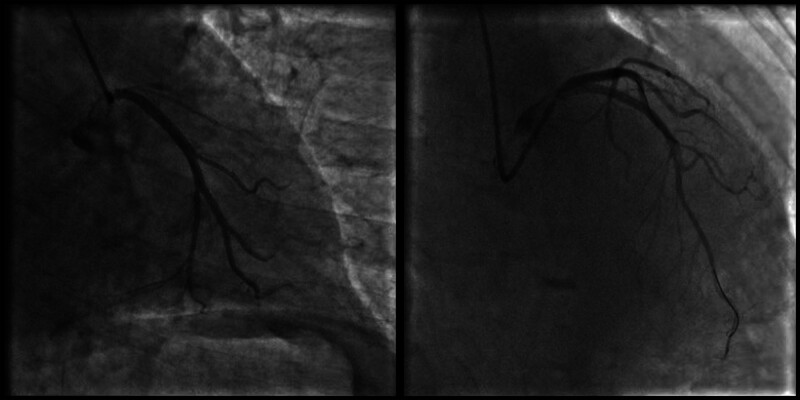
Coronary angiography: the proximal occlusion of the left anterior descending artery was reopened following stent placement.

The patient remained hemodynamically stable during the initial 5-day period. On hospital day 6, she developed recurrent episodes of ventricular tachycardia (VT; Fig. 2), initiated by R-on-T premature ventricular contractions (Fig. 3), which rapidly degenerated into polymorphic ventricular tachycardia and ventricular fibrillation (VF) requiring multiple electrical defibrillations. Serial electrolyte analysis demonstrated potassium 4.63 mmol/L and magnesium 1.05 mmol/L. Concurrent 12-lead electrocardiogram revealed sinus rhythm (86 bpm) with a prolonged QTc interval (480 ms). Antiarrhythmic management was escalated sequentially: lidocaine infusion (0.03 mg/kg/min), amiodarone protocol (150 mg bolus over 10 minutes, followed by 1 mg/min × 6 hours, then 0.5 mg/min × 26 hours), esmolol infusion (150 μg/kg/min). Persistent VT/VF refractory to pharmacotherapy prompted emergency coronary angiography, which identified proximal left anterior descending artery occlusion treated with drug-eluting stent implantation (Fig. 4).

**Figure 5.**
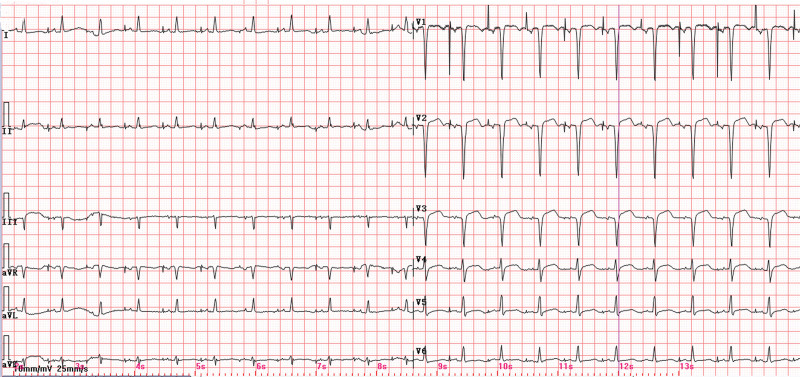
Atrial overdrive pacing at a rate of 95 beats/min.

**Figure 6.**
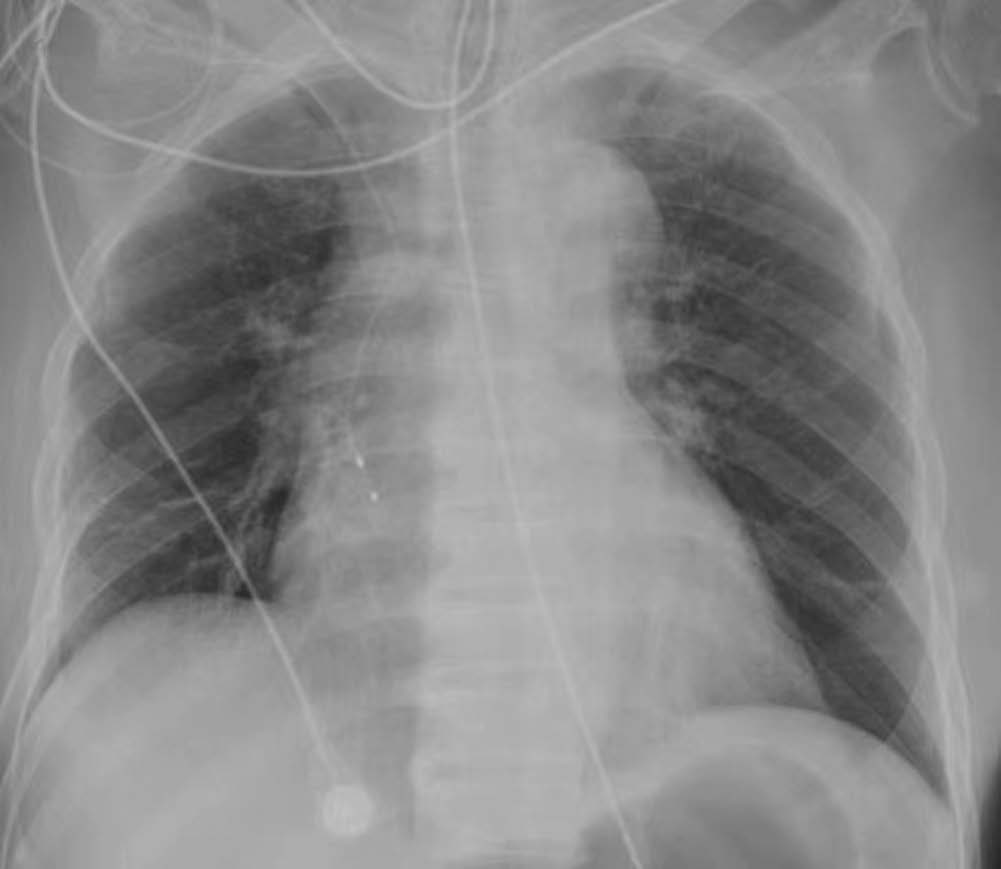
Chest radiograph showing the pacing catheter placed in the right atrium through the subclavian access.

Post-revascularization ventricular arrhythmia recurrence necessitated temporary transvenous overdrive pacing via subclavian access. Serendipitous right atrial appendage lead positioning permitted AOP initiation at 95 bpm (baseline rate 86 bpm, 10–15 bpm above the intrinsic sinus rate; Fig. 5), output of 5 V (2.5 times the capture threshold of 2.0 V), and sensitivity of 0.5 mV. Achieving immediate suppression of ventricular ectopy and arrhythmia termination. All antiarrhythmic agents were discontinued without recurrence, chest radiograph showing the pacing catheter placed in the right atrium through the subclavian access (Fig. 6).

The patient was continuously monitored on telemetry for arrhythmia recurrence, pacing capture, and sensing function. Daily chest X-rays were performed to confirm lead position and screen for complications such as pneumothorax. The access site was examined daily for signs of infection or hematoma.

Continuous holter monitoring after AOP showed no recurrence of ventricular arrhythmias. The temporary pacing lead was electively removed on post-procedure day 5. Serial echocardiographic evaluation demonstrated left ventricular ejection fraction improvement from 40% to 46%, accompanied by NT-proBNP reduction from 33,596 to 9839 pg/mL. Although dual-chamber implantable cardioverter-defibrillator implantation was strongly recommended for secondary prevention, the patient declined due to personal preferences. Long-term antiarrhythmia management consisted of metoprolol tartrate maintenance therapy (12.5 mg twice daily).

During 9-month outpatient surveillance, we follow up with the patient every 2 to 4 weeks using 24-hour holter monitoring, echocardiography, and NT-proBNP levels, no recurrent arrhythmic events or heart failure exacerbations were documented. We will continue long-term follow-up with the patient and will recommend implantable cardioverter-defibrillator implantation based on the risk of sudden cardiac death.

## 3. Discussion

In this case report, we described a patient with AMI who developed ES that could not be controlled with reperfusion and anti-arrhythmic drugs. Subsequently, AOP demonstrated a suppressive effect on the ES.

ES is relatively common after MI. It is currently thought to result from excessive sympathetic activation in the infarcted area, enhanced β-receptor reactivity, and abnormal electrical activity triggered by and conduction related to the Purkinje fiber network at the edge of the infarcted area.^[3]^ It has been shown that MI can lead to an imbalance between sympathetic and parasympathetic tone at the infarct site, which manifests as sympathetic over-innervation and reduced vagal innervation.^[4]^ Moreover, cardiomyocytes in the infarcted area have increased sensitivity to catecholamines and are susceptible to electrical induction of ventricular arrhythmias.^[5]^ Meanwhile, surviving Purkinje fibers across the infarct border zone after MI may exhibit strong autoregulation, triggered activity, and hyper-excitability, which can lead to intraventricular ectopic impulses.^[6,7]^ At the same time, the ectopic electrical impulses of the His-Purkinje conduction system pacing can be transmitted in reverse, blocking atrioventricular conduction and further promoting the recurrence of VT/VF.^[8]^ ES may induce by hypokalemia, hypomagnesemia, intracellular hypercalcemia, acidosis, free fatty acid production due to lipolysis, and free radical production due to reperfusion of the ischemic myocardium.

Half of the cases of sudden cardiac death during the fourth decade of life are related to coronary heart disease, especially malignant arrhythmia caused by coronary artery disease.^[9]^ For patients with recurrent VT after electrical cardioversion, the use of intravenous amiodarone or β-blockers is recommended.^[10]^ But it may increase the QT interval and paradoxically render the VT refractory. In this patient, VT was not controlled despite the aforementioned treatment. After MI, bradycardia is thought to promote ES and is treated with pacing, which is supported by a class IIa recommendation and evidence level C.^[11]^ Slow heart rate may result in a relatively long QT interval. Overdrive ventricular pacing is an effective and readily available treatment for drug-refractory ES caused by bradycardia and the subsequent R-on-T phenomenon. Previous research has shown that temporary transvenous ventricular pacing increases the heart rate and prevents the recurrence of ectopic ventricular beats associated with the acquired long QT syndrome. Therefore, the quantity of proarrhythmic tissue that reaches the refractory period is reduced.^[12]^ But traditional right ventricle endocardial pacing may lead to further hemodynamics deterioration due to worsening left ventricular function.^[13]^ Chalupová et al^[14]^ described a patient who developed drug-refractory ES because bradycardia and long QT after MI, ultimately terminated by AOP.

In this case we reported, she was not experience bradycardia when ES occurred, But the QTc interval was significantly prolonged. Therefore, we consider that the recurrent ES episodes may be related to sympathetic overactivation in the infarcted area, abnormal triggering and conduction of electrical activity in the Purkinje fiber network at the edge of the infarcted area. Meanwhile, it’s also associated with QT interval prolongation leading to an excessively prolonged myocardial vulnerable period. The recurrent episodes of ventricular ES are completely suppressed after AOP. This may be related to the reorganization of atrioventricular forward conduction, inhibition of ventricular ectopic impulse formation, and reflex reduction of cardiac sympathetic tone during AOP.

Crucially, AOP presents superior physiological congruence compared to ventricular pacing modalities. By preserving native atrioventricular synchrony and maintaining the physiological ventricular activation sequence, atrial pacing avoids the deleterious electromechanical discordance inherent in right ventricular endocardial stimulation.^[15]^ This preserved conduction physiology ensures optimal diastolic filling patterns and left ventricular wall motion coordination, thereby enhancing hemodynamic stability – a critical advantage in postinfarction patients with compromised cardiac function. In contrast to ventricular pacing, which induces abnormal depolarization pathways and exacerbates mechanical dyssynchrony,^[16]^ AOP leverages the heart’s intrinsic conduction system to achieve rhythm regularization while preserving normal biventricular activation patterns. Therefore, AOP demonstrates greater superiority compared to ventricular overdrive pacing in the management of ES.

However, bedside temporary atrial pacing electrodes are prone to dislocation. It is recommended to perform active atrial electrode implantation under X-ray guidance.

## 4. Conclusion

Temporary AOP is an effective and safe treatment for resistant ES after AMI.

## Author contributions

**Data curation**: Guo-Li Ma, Li-Na Ji, Ba-Ya-Er Qi.

**Project administration**: Xiao-Ping Liu.

**Writing – original draft**: Xin-Yuan Xu.

**Writing – review & editing**: Rui-Xia He, Hai-Jun Wang, Peng Liu.

## References

[1] Al-Khatib SM Stevenson WG Ackerman MJ. 2017 AHA/ACC/HRS guideline for management of patients with ventricular arrhythmias and the prevention of sudden cardiac death: a report of the American College of Cardiology/American Heart Association task force on clinical practice guidelines and the Heart Rhythm Society. Heart Rhythm. 2018;15:e73–e189. 29097319 10.1016/j.hrthm.2017.10.036

[2] Thygesen K Alpert JS Jaffe AS. Fourth universal definition of myocardial infarction (2018). Circulation. 2018;138:e618–51. 30571511 10.1161/CIR.0000000000000617

[3] Bänsch D Oyang F Antz M. Successful catheter ablation of electrical storm after myocardial infarction. Circulation. 2003;108:3011–6. 14662718 10.1161/01.CIR.0000103701.30662.5C

[4] Kowlgi GN Cha YM. Management of ventricular electrical storm: a contemporary appraisal. Europace. 2020;22:1768–80. 32984880 10.1093/europace/euaa232

[5] D’souza S Saksena S Butani M. Calming the electrical storm: use of stellate ganglion block and thoracic epidural in intractable ventricular tachycardia. Indian J Crit Care Med. 2018;22:743–5. 30405288 10.4103/ijccm.IJCCM_33_18 PMC6201651

[6] Chialvo DR Michaels DC Jalife J. Supernormal excitability as a mechanism of chaotic dynamics of activation in cardiac Purkinje fibers. Circ Res. 1990;66:525–45. 2297816 10.1161/01.res.66.2.525

[7] Berenfeld O Jalife J. Purkinje-muscle reentry as a mechanism of polymorphic ventricular arrhythmias in a 3-dimensional model of the ventricles. Circ Res. 1998;82:1063–77. 9622159 10.1161/01.res.82.10.1063

[8] Arnar DO Bullinga JR Martins JB. Role of the Purkinje system in spontaneous ventricular tachycardia during acute ischemia in a canine model. Circulation. 1997;96:2421–9. 9337219 10.1161/01.cir.96.7.2421

[9] Waldmann V Karam N Bougouin W. Burden of coronary artery disease as a cause of sudden cardiac arrest in the young. J Am Coll Cardiol. 2019;73:2118–20. 31023437 10.1016/j.jacc.2019.01.064

[10] Cardiovascular Branch of Chinese Medical Association. Guidelines for the diagnosis and treatment of acute ST segment elevation myocardial infarction. Chin J Cardiol. 2019;47:766–73.

[11] Ohsawa S Isono H Ojima E. Electrical storm 11 days after acute myocardial infarction: a case report. J Med Case Rep. 2019;13:346. 31771621 10.1186/s13256-019-2267-5 PMC6880439

[12] Maruyama M. Management of electrical storm: the mechanism matters. J Arrhythmia. 2014;30:242–9.

[13] Jiang L Wang L Zhao C. Atrial-His bundle pacing in fulminant myocarditis with ventricular arrhythmia: a case report. BMC Cardiovasc Disord. 2022;22:497. 36418950 10.1186/s12872-022-02936-8 PMC9682640

[14] Chalupová M Suter P Graf D Cook S. Temporary atrial overdrive pacing during a drug-refractory electrical storm in acute myocardial infarction. BMJ Case Rep. 2021;14:e242100. 10.1136/bcr-2021-242100 PMC8420696 34479878

[15] Charlton NP Lawrence DT Brady WJ Kirk MA Holstege CP. Termination of drug-induced torsades de pointes with overdrive pacing. Am J Emerg Med. 2010;28:95–102. 20006210 10.1016/j.ajem.2008.09.029

[16] Miyajima K Urushida T Tamura T. Assessing cardiac mechanical dyssynchrony in left bundle branch area pacing and right ventricular septal pacing using myocardial perfusion scintigraphy in the acute phase of pacemaker implantation. J Cardiovasc Electrophysiol. 2022;33:1826–36. 35748386 10.1111/jce.15609

